# Silent Damage, Delayed Symptoms: A Case of Breast Cancer Radiation–Induced Lumbosacral Plexopathy

**DOI:** 10.3390/reports9010039

**Published:** 2026-01-27

**Authors:** Christian Messina

**Affiliations:** Azienda Sanitaria Provinciale Catania, 95100 Catania, Italy; chry.messina@gmail.com

**Keywords:** radiotherapy, radiation-induced peripheral neuropathy, radiation-induced lumbosacral plexopathy, cancer, case report

## Abstract

**Background and Clinical Significance**: Radiation-induced lumbosacral plexopathy (RILP) is a rare but potentially debilitating complication of radiotherapy, typically affecting patients treated for pelvic malignancies. We report the first documented case of asymmetric RILP following radiotherapy for breast cancer. **Case Presentation**: A 64-year-old woman developed progressive left lower limb weakness, foot drop, and sensory disturbances four years after receiving locoregional radiotherapy extending to the left thoracoabdominal and lumbar areas. Electrophysiological studies revealed an asymmetric sensorimotor axonal neuropathy predominantly involving the left lower limb, without conduction block and sparing the upper limbs, whereas needle electromyography of the lower limbs showed fibrillation potentials, positive sharp waves, and fasciculations in the vastus lateralis, tibialis anterior, and medial gastrocnemius muscles on the left. Magnetic resonance imaging demonstrated edema and contrast enhancement of bilateral L2–L4 nerve roots with paraspinal muscle atrophy. Cerebrospinal fluid analysis showed albuminocytologic dissociation and elevated neurofilament levels. After exclusion of alternative diagnoses, including amyotrophic lateral sclerosis and inflammatory neuropathies, a diagnosis of radiation-induced peripheral neuropathy and RILP was made. The patient’s condition stabilized with physiotherapy and symptomatic treatment. **Conclusions**: This case highlights the need for heightened awareness of RILP as a late complication of breast cancer radiotherapy, underscoring the importance of accurate diagnosis to avoid misclassification and unnecessary treatments. Clinicians should carefully integrate all clinical elements—including a thorough remote medical history—since radiation-related neurological damage may manifest many years after the initial insult.

## 1. Introduction and Clinical Significance

Radiation-induced peripheral neuropathy (RIPN) refers to damage of nerve roots, nerve plexuses, or peripheral nerve trunks resulting from exposure to therapeutic radiation, and is now recognized to present with a range of clinical manifestations depending on which structures are affected [1]. RIPN is a chronic, progressive and usually irreversible disease, often appearing several years after radiotherapy (RT) [1]. Its occurrence is rare but increasing with improved long-term cancer survival [1]. The clinical picture of RIPN is heterogeneous, ranging from isolated cranial nerve injury to brachial (RIBP) or lumbosacral plexopathy (RILP) [1]. RILP is a rare consequence after testicular, uro-gynecological and rectal cancer and Hodgkin’s lymphoma RT, whereas RIBP is often due to breast cancer RT [1]. RILP represents a late-onset neurological complication in patients who undergo pelvic radiotherapy [2,3]. Although uncommon, its recognition has grown in parallel with improved long-term survival among oncologic patients [2,3]. The condition is thought to arise from a combination of persistent inflammatory changes, microvascular compromise, and progressive fibrotic remodeling of irradiated tissues, ultimately leading to a slowly evolving neurotoxic process that may remain clinically silent for years [2,3]. Clinically, RILP can resemble lower motor neuron disorders, typically manifesting with unilateral or bilateral lower-limb weakness, muscle wasting, neuropathic pain, and gradually worsening sensory or proprioceptive impairment [2,3]. Diagnosis relies on a comprehensive clinical history together with targeted investigations—particularly Magnetic Resonance Imaging (MRI) of the lumbosacral plexus and spine, and electrodiagnostic testing—to rule out more common etiologies of plexopathy [2,3]. In current clinical practice, management of RILP remains purely symptomatic [1]. No definitive curative therapy is available. Therefore, the most effective strategy is still prevention, achieved by adhering to radiotherapy dose constraints—reducing total dose, fraction size, and irradiated volume whenever feasible—and by carefully identifying patients with significant comorbidities who may be at higher risk [1]. In this report, we describe the first case of RILP that developed following RT for breast cancer, emphasizing the need for heightened clinical awareness of this late complication to ensure timely recognition, avoid misdiagnosis, and appropriately contextualize prior medical and radiation history.

## 2. Case Presentation

A 64-year-old Italian woman presented with progressive left lower-limb weakness that had started approximately eighteen months earlier and had gradually worsened. Her medical history included hypothyroidism and psoriasis. She denied any additional comorbidities associated with peripheral neuropathy, such as diabetes mellitus, metabolic syndrome, previous alcohol or substance abuse, vitamin deficiencies, or a family history of polyneuropathies or gait disorders. Her oncological history was notable for a left-sided breast cancer located in the lower quadrants of the breast, below the nipple; the patient also presented with bilateral breast ptosis. In 2021, she underwent surgery followed by adjuvant locoregional radiotherapy delivered using conventional 3D conformal radiotherapy (3D-CRT) with a linear accelerator system. The treatment was planned through the institutional treatment planning system and consisted of a total dose of 50–50.4 Gy administered in 25–28 fractions (1.8–2 Gy per fraction) five days per week, targeting the left breast and regional lymphatic areas. No direct pelvic irradiation was performed. Unfortunately, the original detailed treatment planning data, equipment specifics, and dosimetric data for the lumbosacral plexus were not available for review due to archival limitations. Beginning in January 2024, she developed insidious left foot pain and paresthesias, followed by foot drop, which led to repeated falls and fractures of the left foot. Neurological examination revealed marked atrophy and weakness in the left lower limb, predominantly distally. Muscle strength according to the MRC scale was 2/5 in the left extensor digitorum brevis and tibialis anterior, 4/5 in the quadriceps femoris, and 5/5 in the remaining left-sided muscle groups. On the right, strength was 4/5 in the tibialis anterior and extensor digitorum brevis, and otherwise preserved. A mild reduction in light-touch sensation was observed over the left leg. Nerve conduction studies (NCS) revealed an asymmetric sensorimotor axonal neuropathy of the lower limbs, more pronounced on the left, without evidence of conduction blocks. To evaluate possible involvement of peripheral nerves or nerve roots in the upper extremities, both NCS and needle electromyography (EMG) were extended to the upper limbs during the initial and follow-up assessments; however, no abnormalities were detected in the upper limbs in either study. EMG of the lower limbs showed fibrillation potentials, positive sharp waves, and fasciculations in the vastus lateralis, tibialis anterior, and medial gastrocnemius muscles on the left. These electrophysiological findings were confirmed and remained stable in a repeat NCS/EMG examination performed six and twelve months later. Whole-spine MRI demonstrated normal cervical and thoracic segments, while the lumbar study revealed edema of the bilateral L2–L3–L4 nerve roots, with contrast enhancement of the left-sided roots, and asymmetric paraspinal muscle atrophy. Lower-limb Doppler ultrasound showed no evidence of vascular or lymphatic disease. Blood tests demonstrated mildly elevated anti-thyroperoxidase antibodies and ANA 1:80 (speckled pattern), but normal levels of serum glucose, vitamin B12, creatinine, total proteins, electrophoresis, cryoglobulins, anti-ganglioside and anti-neuronal antibodies, tumoral markers, serum folates, iron indices, and infectious serologies—including HIV, viral hepatitis, herpesviruses, Brucella, and Mycobacterium leprae. Autoimmune screening was negative for ENA panel, c-ANCA, p-ANCA, and anti-CCP antibodies. Genetic testing for transthyretin amyloidosis and Charcot–Marie–Tooth disease was also normal. Cerebrospinal fluid (CSF) analysis revealed marked albuminocytologic dissociation (ACD) and significantly elevated neurofilament levels (light chain: 11,800 pg/mL; heavy chain: 2.67 ng/mL). The patient subsequently received corticosteroid therapy followed by a five-day course of intravenous immunoglobulins (IVIg), aimed at reducing both inflammation and edema, as well as serving a diagnostic purpose to include or exclude an inflammatory/autoimmune etiology. However, these treatments did not yield meaningful clinical improvement, shifting away from the hypothesis of an inflammatory pathogenesis. A second neurological examination performed two months after the initial assessment showed no further deterioration. Additional follow-up evaluations at six and nine months likewise demonstrated clinical stability without new deficits or progression. Other potential causes such as paraneoplastic neuropathy and radiation-associated tumor recurrence were also considered and ruled out. Paraneoplastic neuropathy was deemed unlikely due to the absence of systemic symptoms, negative onconeural antibody testing, and lack of clinical progression typical of paraneoplastic syndromes. Repeated imaging examinations showed no evidence of tumor recurrence or local-regional disease, effectively excluding radiation-associated tumor regrowth as a cause of the patient’s symptoms. Given the absence of alternative etiologies and the temporal relationship with previous RT, the overall clinical, electrophysiological, and radiological findings were consistent with a RILP secondary to breast cancer RT. The patient currently remains clinically stable, which is attributed to ongoing physiokinesitherapy administered three times per week, performed to prevent further deterioration of mobility and to stimulate movement. Additionally, her sensory symptoms are well controlled with carbamazepine at a dose of 1200 mg per day, used to reduce neuropathic pain, alongside supplementation with B-complex vitamins.

## 3. Discussion

To the best of our knowledge, RILP has not previously been described as a complication of standard adjuvant RT for breast cancer. RT causes both direct and indirect cellular injury, damaging DNA and impairing essential intracellular processes [4]. High-energy radiation generates reactive oxygen species, which trigger cascading oxidative reactions that further disrupt cellular integrity [4]. Rapidly proliferating tissues—such as tumor cells, mucosal layers, skin, and hair follicles—are particularly vulnerable to this acute toxicity, whereas healthy cells may partially recover through intrinsic repair pathways [4]. Nonetheless, radiation-induced inflammation can lead to apoptosis even in non-cancerous tissues [4]. Neurons, being post-mitotic, have limited ability to repair radiation-related insults, and their clinically relevant manifestations often emerge as chronic rather than immediate effects [4]. Ongoing oxidative stress, microvascular damage, and progressive fibrosis contribute to neural injury and Wallerian degeneration, placing radiation-induced plexopathy within the broader spectrum of radiation fibrosis syndrome [4]. The risk of such complications increases with higher cumulative doses, extended treatment times, larger irradiation fields, and concomitant chemotherapy [4]. The pathophysiology of radiation-induced injury to nerve roots and peripheral nerves is partly attributable to initial microvascular injury, followed by radiation-induced fibrosis (RIF) [1]. It has been shown that it is characterized by an early asymptomatic prefibrotic phase with chronic inflammation, then a reversible and organized fibrotic phase of extracellular matrix deposits, and a late irreversible fibroatrophic poorly vascularized phase with retractile fibrosis [1]. Moreover, it seems that peripheral nerves are quite sensitive to RT damage [1]. Direct effects of RT on nerve include bioelectrical alterations (subnormal action potentials, altered conduction time), enzyme changes, abnormal microtubule assembly, altered vascular permeability and neurilemmal damage, which are observed experimentally within 2 days after irradiation and are all dose-dependent and irreversible [5]. Furthermore, indirect effects of RT are mainly vascular not only on the nerve more interested, but on the other adjacent peripheral nerve [5]. Radiation does not affect all segments of the spinal cord uniformly. The lower cervical cord is relatively shielded by surrounding bone and connective tissue, whereas the anterior portions of the lower thoracic and upper lumbar cord lack comparable anatomical protection [1]. Nonetheless, the extent of radiation-induced injury depends not only on the dose, but also on beam orientation, field selectivity, tissue density, and the traversal characteristics of the irradiated structures [1]. Clinical manifestations may emerge months to decades after exposure [1]. Some authors have proposed that RT can preferentially damage lower motor neurons within the spinal cord, whereas others argue that the spinal roots or peripheral nerves represent the primary targets of injury [1]. Patients with RILP often develop early symptoms characterized by low back discomfort radiating toward the proximal thigh, accompanied by unilateral paresthesias and progressive weakness predominantly involving the distal lower limb within the lumbar trunk territory [3,4]. RILP primarily involves the upper components of the lumbosacral plexus, particularly the L2–L4 nerve roots, and the corresponding motor and sensory deficits generally follow well-defined anatomical patterns reflecting the affected segments [3,4]. While pain may precede neurological deterioration by several weeks or months, only a subset of individuals (10–33%) with RILP report significant pain, and when present it is generally mild and neuropathic in quality, with manifestations such as paresthesias, hyperalgesia, or allodynia [3,4]. A classic physical finding is foot drop, reflecting involvement of the lumbosacral trunk [3,4]. In more advanced cases, patients may also experience urinary or fecal incontinence, potentially related to neurogenic dysfunction of the bladder or bowel [3,4]. Nevertheless, radiation-related neurotoxicity is likely the result of simultaneous and cumulative effects across multiple levels of the nervous system [1]. It seems that 40 Gy is a sufficient dose to cause peripheral nerve damage, where clinical onset could range of 0.4–25 years after external RT [1]. Although detailed dosimetric data for the lumbosacral plexus were not available in this case, the literature suggests that the tolerance dose of the lumbosacral plexus (defined as a 5% probability of severe sequelae within 5 years) ranges between 47 and 60 Gy when RT is administered alone or combined with brachytherapy, respectively [6]. Radiation-induced lumbosacral plexopathy (RILSP) has been reported with doses as high as 70–80 Gy for full-volume irradiation [6]. However, peripheral nerve radiosensitivity is likely increased by concomitant chemotherapy, with cases of RILSP described at doses as low as 50–60 Gy [6]. Thus, the lumbosacral plexus is considered an organ at risk during intensity-modulated radiotherapy (IMRT) planning [6]. An important dosimetric study demonstrated that, when the lumbosacral plexus is contoured and evaluated in RT plans for pelvic tumors, the incidence of RILSP can be approximately 7–8% in patients receiving doses within this range [6]. In our patient, the prescribed total dose was 50 Gy, below these thresholds, but indirect effects such as radiation scatter, microvascular injury, and fibrosis may contribute to plexus damage even at lower doses. Anatomically, the human breast is typically located between the second and seventh ribs. However, in this patient, bilateral breast ptosis and the tumor’s location in the lower quadrants beneath the nipple likely led to an inferior translation of the radiation field. Consequently, the irradiated area was probably extended below the thoracic cage, exposing the upper lumbar spinal nerve roots, which are not protected by the rib cage. This anatomical consideration supports the hypothesis that the lumbosacral plexopathy observed may be related to unintended radiation exposure of the upper lumbar nerve roots. During RILP, the neurological deficits are bilateral and asymmetric with initial unilateral damage and largely motor [1], like in our described case, where onset and symptoms severity started from the left lower limb, which was the same side of precedent RT. RILP diagnosis might be difficult because of its nonspecific clinical and radiological features [1]. In cases presenting with predominantly motor involvement, the principal differential diagnosis is amyotrophic lateral sclerosis (ALS) [1]. However, several elements in our patient strongly argued against ALS. She exhibited a sensorimotor axonal neuropathy, rather than a pure motor neuron disorder; MRI demonstrated edema of the bilateral lumbar nerve roots with contrast enhancement on the left, with no involvement of cervical or thoracic spinal segments; and follow-up examinations showed minimal progression over time, which is atypical for ALS. Additionally, no upper motor neuron signs were detected at any stage, further excluding a diagnosis of ALS [7]. ACD in CSF can be indicative of inflammatory polyneuropathies such as Chronic Inflammatory Demyelinating Polyneuropathy (CIDP). However, it has been demonstrated that ACD is not specific to inflammatory neuropathies; toxic or non-inflammatory neuropathies may also present with elevated CSF protein levels [8]. A previous case report documented elevated CSF protein levels following neurosurgical intervention and RT performed for the resection of an astrocytoma, suggesting that such treatments themselves may contribute to CSF protein abnormalities [9]. Furthermore, the patient’s lack of clinical improvement following corticosteroid and IVIg therapy argues against an inflammatory etiology. Our hypothesis is that the ACD results from blood–brain barrier disruption and immune system activation triggered by exposure to neuronal antigens. The increased protein concentration in the CSF may arise from multiple mechanisms, including post-radiation tissue necrosis, leakage of proteins from damaged capillaries, inflammation, and increased vascular permeability. Elevated neurofilament levels in CSF may prompt consideration of a neurodegenerative process. However, neurofilament proteins are now recognized as sensitive biomarkers of neuro-axonal injury across a broad spectrum of neurological disorders, including those that are not neurodegenerative in nature [10]. In a previous study, irradiated mice subjected to cranial radiation exhibited significantly increased neurofilament light chain (NFL) levels [11]. Similarly, serum NFL has emerged as a reliable biomarker for neuronal injury, with its concentration unaffected by blood–brain barrier permeability [11]. Moreover, elevated CSF NFL concentrations were observed for three months following prophylactic cranial radiotherapy in patients with small-cell lung cancer (SCLC), supporting its role as a marker of radiation-induced neuronal damage [12]. Based on these observations, we hypothesize that RT administered for breast cancer in this case contributed to the development of RILP. Notably, the radiation field extended beyond the thoracic region into the lumbar area, which likely resulted in direct damage to the lumbar plexus. This broader exposure may explain the localization of the neuropathic symptoms and the electrophysiological and radiological findings observed in the patient. However, management of radiation-induced plexopathy or polyneuropathy focuses mainly on symptom relief, given the progressive and often irreversible nature of the condition [3,4]. Neuropathic pain and sensory disturbances can be treated with topical agents (e.g., capsaicin), common analgesics like acetaminophen and ibuprofen, and prescription drugs including gabapentin, pregabalin, duloxetine, amitriptyline, carbamazepine, or opioids [4]. Physical therapy and gait rehabilitation are essential to strengthen lower limbs, enhance mobility, and determine the need for assistive devices such as canes or orthoses [4]. Additionally, patients with RILP frequently develop lymphedema, which may require ongoing management through manual lymphatic drainage, compression therapy, and pneumatic pumps [4]. Given that this report describes a single case, causal inference must be regarded as indirect. The absence of direct dosimetric data specific to the lumbosacral plexus further limits definitive conclusions. Therefore, the generalizability of these findings is restricted. Nevertheless, the clinical presentation, comprehensive diagnostic work-up, and exclusion of alternative etiologies provide strong support for the diagnosis of RILP in this patient.

## 4. Diagnostic Challenges

Diagnosing RILP is complex due to its nonspecific clinical presentation and the absence of definitive diagnostic markers. A major challenge is the prolonged latency period; symptoms can manifest years or even decades after RT, making the causal link difficult to establish. This highlights the critical importance of a detailed clinical history to identify prior radiation exposure. The clinical features of RILP overlap with many more common disorders, including neoplastic plexopathy, lumbar radiculopathies, and peripheral neuropathies. Therefore, a comprehensive differential diagnosis must exclude metabolic, endocrine, inflammatory, genetic, compressive, infectious, and autoimmune causes. Imaging with MRI plays a fundamental role but may be limited by post-radiation fibrosis obscuring normal tissue planes and complicating differentiation from tumor recurrence. Electrodiagnostic studies can support the diagnosis but often lack specificity, necessitating cautious interpretation. Consequently, diagnosis is frequently delayed or initially misattributed. In our case, the diagnostic process was further complicated by the atypical inferior extension of the radiation field due to the patient’s anatomical features, which likely resulted in unexpected exposure of the lumbosacral plexus. This unusual radiation distribution made clinical assessment and diagnosis particularly challenging.

## 5. Conclusions

To our knowledge, this represents the first reported case of RILP following breast cancer RT. However, it is plausible that many other cases remain misdiagnosed, particularly when the radiation field extends beyond the expected anatomical regions. Radiation-induced nerve damage can manifest many years after treatment, underscoring the importance of thoroughly investigating remote oncological histories during clinical evaluation. Even neurological symptoms arising long after RT should prompt consideration of prior radiation exposure as a contributing factor, as pre-existing subclinical damage may predispose patients to late-onset neuropathies. Recognizing this association is crucial to avoid unnecessary and potentially harmful treatments—often costly and associated with adverse effects—and to guide clinicians toward the most appropriate management strategies aimed at preventing further neurological deterioration in affected patients. Ultimately, heightened awareness and early recognition of RILP are essential steps toward improving patient outcomes and quality of life in cancer survivors.

## Data Availability

No new data were created.
