# Expression of Concern: Messina, C. Silent Damage, Delayed Symptoms: A Case of Breast Cancer Radiation–Induced Lumbosacral Plexopathy. *Reports* 2026, *9*, 39

**DOI:** 10.3390/reports9030243

**Published:** 2026-07-27

**Authors:** Reports Editorial Office

**Affiliations:** MDPI, St. Alban-Anlage 66, 4052 Basel, Switzerland; reports@mdpi.com

The Reports Editorial Office and the Editor-in-Chief would like to inform readers of concerns regarding potential scientific irregularities identified in this case report [1]. Following publication, concerns were raised with the Editorial Office regarding the accuracy of the reported data and the interpretation of the findings.

The Editor-in-Chief has decided to issue this expression of concern while an investigation is being conducted. The author was contacted for further clarifications regarding this publication. Once this process is complete, the Editorial Office will provide an update on this situation and announce any post-publication modification deemed to be necessary, as per MDPI’s Updating Published Papers policy.
